# Determinants of Bird Richness in the Louzishan National Nature Reserve: Effects of Productivity and Habitat Heterogeneity Across Taxonomic and Functional Dimensions

**DOI:** 10.1002/ece3.73860

**Published:** 2026-06-17

**Authors:** Xiaocui Ma, Meiting Wang, Shiguang Zhang, Dongmei Wan, Yiting Jiang

**Affiliations:** ^1^ Laboratory of Animal Resources and Epidemic Prevention School of Life Sciences Liaoning University Shenyang China

**Keywords:** beta diversity, community composition, forest community, habitat heterogeneity

## Abstract

Human activities are modifying Earth's ecosystems in different ways and at different scales, and conserving multiple aspects of biodiversity has become important for maintaining ecosystem stability. Plant productivity and habitat heterogeneity are important environmental factors for bird survival. In this study, we investigated the impact of these two factors on avian taxonomic and functional diversity in the Louzishan National Nature Reserve, a small protected area in Liaoning Province, China. We surveyed bird communities during summer (breeding season) and winter (resident period) and used NDVI as a proxy for productivity, while habitat heterogeneity was quantified based on landscape composition. We then analyzed taxonomic and functional alpha diversity (Shannon index and Rao's Q) and beta diversity (β_sor_, β_sim_, β_sne_) in relation to productivity, habitat heterogeneity, and spatial distance. Our results revealed complex seasonal patterns. In summer, pairwise differences in NDVI were positively related with taxonomic beta diversity (β_sor_) and its turnover component (β_sim_), with turnover dominating total beta diversity, indicating that species replacement driven by productivity gradients is the main mechanism. Summer pairwise differences in habitat heterogeneity were positively related with the nestedness component (β_sne_). In summer, NDVI was positively related with taxonomic and functional alpha diversity. In winter, habitat heterogeneity showed a significant negative relation with taxonomic alpha diversity but a hump‐shaped (nonlinear) relationship with functional alpha diversity; pairwise differences in NDVI and habitat heterogeneity were not significantly related to any winter beta diversity component, whereas spatial distance was positively related with both overall taxonomic and functional beta diversity. Overall, our findings highlight a seasonal shift in community assembly processes: environmental filtering (productivity and habitat heterogeneity) dominates in summer, whereas dispersal limitation prevails in winter. These results underscore the importance of considering seasonal dynamics and multiple diversity dimensions when assessing the conservation value of small protected areas.

## Graphical abstract

We want to reveal the relationship between the alpha and beta diversity of birds and its two components with vegetation productivity and habitat heterogeneity from both taxonomic and functional perspectives. Our findings reveal productivity, as an important environmental variable, influences bird species richness and compositional differences from two dimensions. A homogeneous forest is more conducive to maintaining the diversity of bird communities than disturbed complex habitats. Even if there is a high level of species turnover in the region, the functions of these species may be homogeneous and redundant, which is not beneficial to improve ecosystem resilience.

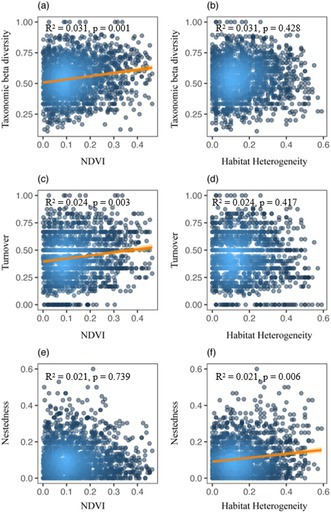

## 1 Introduction

A central objective in ecology and conservation biology is to understand the spatial assembly patterns of biological communities and to elucidate the key mechanisms driving biodiversity distributions (Ding et al. 2021; Hanz et al. 2019; Sutherland et al. 2013). Human activities, through habitat loss and fragmentation, have exerted pronounced negative impacts on biological communities and biodiversity in many regions, even threatening local ecosystem functioning and stability (Sol et al. 2020). Although a global network of protected areas, such as national parks and nature reserves, has been established to collectively safeguard biodiversity, differences in the spatial structure of biological communities between protected areas and their surrounding landscapes, as well as the underlying mechanisms shaping their respective biodiversity patterns, remain poorly understood.

Currently, most studies measure alpha diversity at a regional scale to reflect the spatial assembly characteristics of biological communities. When comparing different communities, alpha diversity primarily focuses on the level of biodiversity within a specific site, habitat, or local community (Clavel et al. 2011; Coetzee and Chown 2016). In contrast, beta diversity can characterize the differences or turnover rates in biodiversity composition between different communities, revealing how species composition changes along environmental gradients or across different habitats (Marcacci et al. 2022). For example, when intensive land use leads to an overall decline in the abundance of most species within a community, the presence of rare species in the community may still drive an increase in beta diversity among communities, even if species are lost (Karp et al. 2012). Turnover occurs when existing species are replaced by different species in new locations; nestedness arises when the loss or gain of species results in species‐poor sites being strict subsets of species‐rich sites (Baselga 2010). That is, two ecological mechanisms—niche differentiation and environmental filtering—drive turnover; while processes such as selective extinction, selective immigration, and habitat nestedness lead to differences in species richness among communities and thus generate nestedness (Baselga 2010; Si et al. 2016). Therefore, decomposing beta diversity is an important approach for elucidating the core mechanisms that influence biodiversity patterns.

Productivity gradients influence community assembly processes by modulating patterns of resource competition (Haedo et al. 2017). Under low productivity conditions, resource scarcity intensifies environmental filtering, resulting in the persistence of only a few species adapted to nutrient‐poor environments. Niche theory posits that species coexistence depends on the differentiation of resource utilization (niche differentiation) and differences in species' environmental tolerances (Kneitel 2019). The habitat heterogeneity hypothesis further elucidates that areas with higher environmental heterogeneity can provide a greater diversity of niches, thereby promoting species coexistence through niche differentiation. In regions with low habitat heterogeneity, environmental conditions tend to be homogenized, which leads to the gradual disappearance of species unable to adapt to the environment, thus becoming the main source of differences between different communities (MacArthur and MacArthur 1961). Therefore, productivity and habitat heterogeneity, as key factors regulating the spatial distribution and total amount of resources, are regarded as two fundamental pillars driving the spatial assembly characteristics of biological communities. Since productivity and habitat heterogeneity can promote species coexistence within communities by increasing total resource availability and niche space (Haedo et al. 2017). This study expects that alpha diversity increases with rising productivity and habitat heterogeneity, and that the turnover component of beta diversity increases with increasing differences in productivity and habitat heterogeneity among sites.

Although these mechanisms have been partially validated at the taxonomic diversity level (Evans et al. 2005), how functional diversity responds to productivity and habitat heterogeneity still varies across different ecosystems or research scales. The Louzishan National Nature Reserve is located in Kazuo County, Chaoyang City, Liaoning Province. Compared with large national parks and other extensive protected areas, it is a relatively small protected area (Zhang 2019). The reserve has a variety of habitat types, providing an ideal platform for testing the relative contributions of productivity and habitat heterogeneity to avian diversity. Birds, due to their strong dispersal capacity across different habitats, rich diversity in body size, highly specialized behaviors and dietary traits, and rapid response to environmental changes, have become a widely used indicator group for ecological assessments (Thorn et al. 2018). A comprehensive analysis of bird diversity from both taxonomic (Villaseñor et al. 2021) and functional (Jarzyna and Jetz 2018) dimensions contributes to a deeper understanding of the spatial assembly characteristics of biological communities and their driving mechanisms (Coetzee and Chown 2016).

This study was conducted within the reserve and its surrounding areas, aiming to elucidate the relationship between the spatial structure of biotic communities in a small protected area and its surrounding landscape, and to understand the significance of small protected areas for biodiversity conservation.

## 2 Materials and Methods

### 2.1 Study Area

**FIGURE 1:**
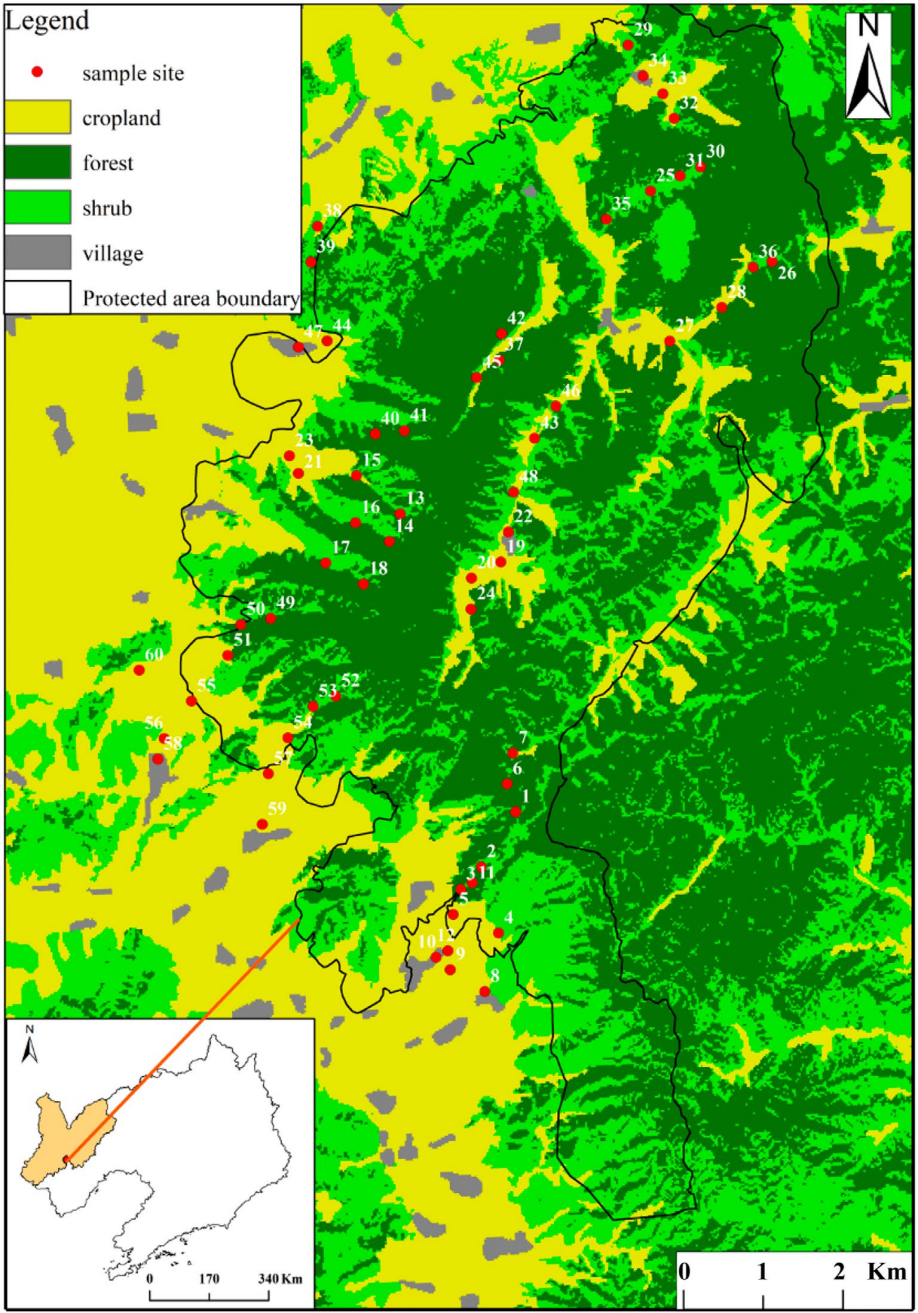
Schematic map of the study area and survey sites.

This study focuses on the Louzishan National Nature Reserve (41°0′20″–41°11′20″ N, 119°54′50″–120°2′50″ E, Figure 1) and its surrounding areas. The reserve is located in the middle section of the Songling Mountains, which extend eastward from the Yanshan Mountains, belonging to the hilly and low mountainous region of the Hebei–Liaoning border. It covers a total area of approximately 111.5 km^2^. The reserve lies in a temperate, semi‐humid, and semi‐arid continental monsoon climate zone. Spring is dry with little rainfall and frequent strong winds; summer is hot and rainy; autumn features clear skies and crisp air; winter is cold and dry. From June to August, the average temperature is 23.7°C, the precipitation is 344.5 mm, accounting for 67.9% of the annual precipitation, and the sunshine duration is 679.2 h. Winter is cold and dry: from November to February of the following year, the average temperature is −4.9°C, the average winter precipitation is 14.7 mm, accounting for 2.9% of the annual precipitation, and the sunshine duration is 779.8 h. The vegetation type falls under the warm‐temperate deciduous broad‐leaved forest, with vegetation formation groups including coniferous forest, broad‐leaved forest, shrubland, and shrub‐grassland. It should be noted that, like most nature reserves in China, the Louzishan National Nature Reserve implements a zoning management system consisting of core, buffer, and experimental zones. Anthropogenic landscape elements such as agricultural land, scattered villages, and paved roads (impervious surfaces) are mainly distributed in the experimental zone and the areas surrounding the reserve.

### 2.2 Bird Survey

The bird survey was conducted in winter (November 2022 and February 2023) and summer (May–June 2023). 60 survey sample sites were selected within the study area through random sampling and relocated to accessible sites if the original sites were inaccessible geographically. All final sites were at least 200 m apart (Morelli et al. 2013). Birds in each site were surveyed twice per season using standardized point counts. The final abundance data of each bird at each location is the maximum of the results of two surveys. We recorded all forest birds seen or heard within a radius of 50 m from the sample point, and each point count lasted 10 min (excluding waterfowl and nocturnal birds). Surveys were conducted under favorable weather conditions. We plotted the species accumulation curve and calculated sample coverage at each site to ensure that surveys were adequate. The species accumulation curve was plotted using the function “specaccum” in the “vegan” package (Dixon 2003), and sample coverage was calculated using the “iNEXT” function in the “iNEXT” package (Hsieh et al. 2016).

### 2.3 Functional Traits

To quantify bird functional diversity, body mass, main feeding guild, and main foraging strata were selected as functional traits (Ding et al. 2013). All trait data were collected from Birds of the World (Dunn et al. 2020). All species were classified into feeding guilds based on their preferred food resource (seed, invertebrate, vertebrate, omnivore). Their foraging behaviors were characterized based on their optimal foraging strata (ground, understory, medium, canopy, aerial). All variables, except body mass, were binomial (scored as either 0 or 1). First, we calculated the functional distance between all species pairs according to their trait values using Gower's distance (Gower 1971), which supported the mixing of different types of variables. We then performed principal coordinate analysis (PCoA), retained the first two axes (87% of total inertia), and used them in the following analysis. We calculated Gower's distances with the function “gowdis” in the “FD” package (Laliberté and Legendre 2010), and PCoA with the function “cmdscale” in the “stats” package.

### 2.4 Environmental Variables

The Normalized Difference Vegetation Index (NDVI) is a widely used indicator of net primary productivity. It can be used as a proxy for resource availability for birds (Benedetti et al. 2023). We calculated NDVI using Landsat 8 OLI_TIRS remote sensing images (30 m), which were downloaded from the Geospatial Data Cloud site, Computer Network Information Center, and the Chinese Academy of Sciences (http://www.gscloud.cn). As satellite images for 2022 and 2023 were not available, and there was no significant change in land use over these 2 years, images corresponding to the survey time in 2021 were used instead. Atmospheric correction and radiometric calibration of the satellite images were performed in ENVI 5.3 (Harris Geospatial Solutions 2016). NDVI was calculated using the band math tool [(NIR Red)/(NIR + Red)]. Subsequently, ArcGIS 10.2 was used to calculate the mean NDVI within a buffer zone of 200 m around the sample points.

Land cover map (30‐m spatial resolution) of the study area was downloaded from the Globalland30 dataset (http://www.globallandcover.com). The map was used to estimate the proportional shares of impervious and sealed surfaces (such as roads and buildings), forests, shrubs, and fields. Based on their proportions we calculated habitat heterogeneity within a 200 m radius centered on 60 sample points (Simpson's diversity index).

### 2.5 Alpha and Beta Diversity Metrics

Birds' summer and winter communities were assessed.

Taxonomic α‐diversity was measured using the Shannon–Wiener index, and functional α‐diversity was measured using Rao's quadratic entropy (RaoQ) (Morris et al. 2014; Mouchet et al. 2010). These alpha diversity calculations were performed using packages “vegan” and “FD”.

For beta diversity analysis, we used pairwise Sørensen dissimilarities (incidence‐based pairwise dissimilarities) and functional pairwise Sørensen dissimilarities (incidence‐based pairwise functional trait space dissimilarities). Sørensen dissimilarity (β_sor_) represents total beta diversity and can be partitioned into two additive components, namely species turnover (β_sim_) and nestedness (β_sne_). In this study, β_sor_ and β_funcsor_ represent the value of the total beta diversity of taxonomic and functional beta diversity, β_sim_ and β_funcsim_ represent the turnover component of taxonomic and functional beta diversity, and β_sne_ and β_funcsne_ represent the nestedness component of taxonomic and functional beta diversity. To estimate the overall beta diversity of bird communities among all sample sites, multiple‐site dissimilarity was used. Overall multiple‐site Sørensen dissimilarity (β_SOR_) and multiple‐site Sørensen functional dissimilarity (β_funcSOR_) were measured using multiple‐site Sørensen dissimilarity, which was decomposed into spatial turnover (β_SIM_ and β_funcSIM_) and nestedness components (β_SNE_ and β_funcSNE_) afterward. To determine the respective contribution of turnover and nestedness to overall beta diversity (multiple‐site dissimilarity), we used beta‐diversity ratio: β_ratio_ = β_SIM_/β_SOR_ and β_funcratio_ = β_funcSIM_/β_funcSOR_. β_ratio_ > 0.5 indicates that total beta diversity is determined dominantly by species turnover. The beta diversity was calculated using the “beta. Pair”, “beta.multi”, “functional. Beta. Pair” and “functional.beta.multi” functions in the “betapart” package (Baselga and Orme 2012).

### 2.6 Data Analyses

This study used the Nestedness metric based on Overlap and Decreasing Fill (NODF; Almeida‐Neto et al. 2008) to complement the beta diversity analysis and effectively quantify taxonomic and functional nestedness. To assess the statistical significance of the observed nestedness patterns, a null model that fixed row and column sums was employed to test whether the observed values were significantly higher than random expectations. If *p* < 0.05 and the observed value was greater than the random expectation, the observed network was considered to exhibit significant nestedness relative to the random level. Conversely, if *p* < 0.05 and the observed value was lower than the random expectation, the structure was interpreted as significantly anti‐nested (significance level α=0.05).

We constructed ordinary least squares (OLS) linear regression models of alpha diversity against environmental factors. In this analysis, to ensure the assumption of residual independence in subsequent regression analyses, we tested for spatial autocorrelation in taxonomic alpha diversity, functional alpha diversity, and each environmental factor separately using the global Moran's I statistic, implemented with the spdep R package (Bivand and Wong 2018). The Moran's I test was conducted under the randomization assumption. If the residuals were significant (*p* < 0.05), indicating the presence of spatial structure not captured by the model, we introduced a nonlinear spatial model—the generalized additive model (GAM)—to account for the spatial autocorrelation, and the GAM results were used for interpretation.

Generalized linear model was used to examine the relationships between taxonomic and functional diversity with productivity and habitat heterogeneity. Landscape structure distance matrix was constructed based on Euclidean distances calculated using the ‘vegdist’ function. The geographic distance matrix was derived by calculating pairwise distances between study sites. Multiple regression analysis on matrices analysis (MRM) (Lichstein 2007) was used to explore the relative contribution of productivity and landscape heterogeneity to taxonomic and functional beta diversity and its two components. The MRM analysis was carried out with the ‘MRM’ function in the “ecodist” package (Goslee and Urban 2007) for R. All statistical analysis were performed with R4.5.3 (R Core Team 2025).

## 3 Result

Across summer and winter, 3680 individuals from 49 bird species (28 families, Table S1) were recorded, with 1582 individuals from 35 species in 23 families in summer and 2098 individuals from 35 species in 19 families in winter. The upward trend of the species accumulation curves based on the number of samples was smooth, followed by a gradual asymptote, indicating that the sampling volume of the survey was sufficient (Figure S1). In addition, the data show that in summer, the sample coverage of 33 sampling points is greater than 90% (the observed species to which the observed individuals belong account for more than 90% of the estimated total number of species), 15 sampling points are greater than 80%, 8 sampling points are greater than 70%, and 4 sampling points are greater than 60%. In winter, the sample coverage of 40 sampling points is greater than 90%, 13 sampling points are greater than 80%, 5 sampling points are greater than 70%, and 2 sampling points are greater than 65%. It can also be considered that this bird survey is relatively comprehensive (Table S2).

**FIGURE 2:**
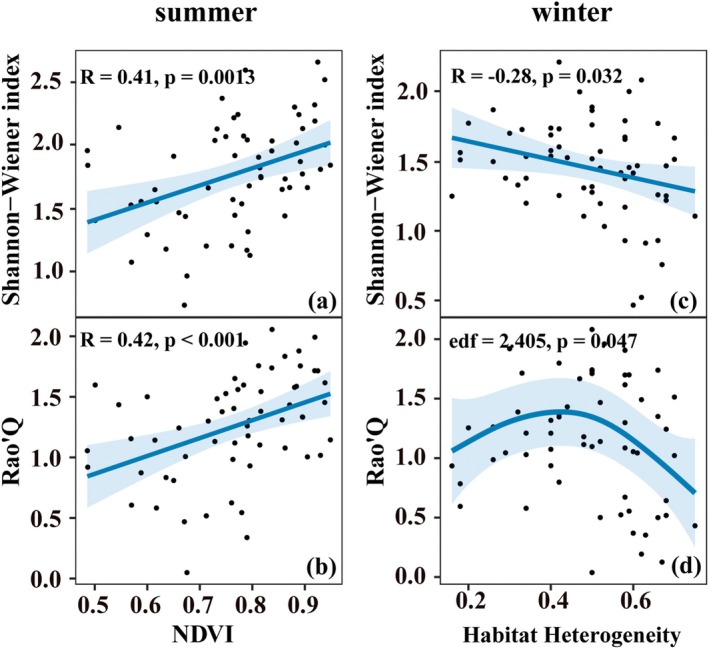
Density scatter plots of (a) summer taxonomic diversity (Shannon–Wiener index) vs. summer NDVI, (b) summer functional diversity (Rao'Q) vs. summer NDVI, (c) winter taxonomic diversity vs. winter habitat heterogeneity, and (d) winter functional diversity vs. winter habitat heterogeneity, based on OLS regression (a–c) and GAM (d). Red fitted lines represent the linear (a–c) and nonlinear (d) relationships derived from the corresponding models.

According to the OLS model results, summer NDVI showed a significant positive relation with taxonomic alpha diversity; winter habitat heterogeneity was significantly negatively related with taxonomic alpha diversity. Summer NDVI also exhibited a significant positive relation with functional alpha diversity. According to the GAM model results, winter habitat heterogeneity had a significant nonlinear effect on functional alpha diversity, whereas the effects of NDVI and spatial distance were not significant (Figure 2).

**FIGURE 3:**
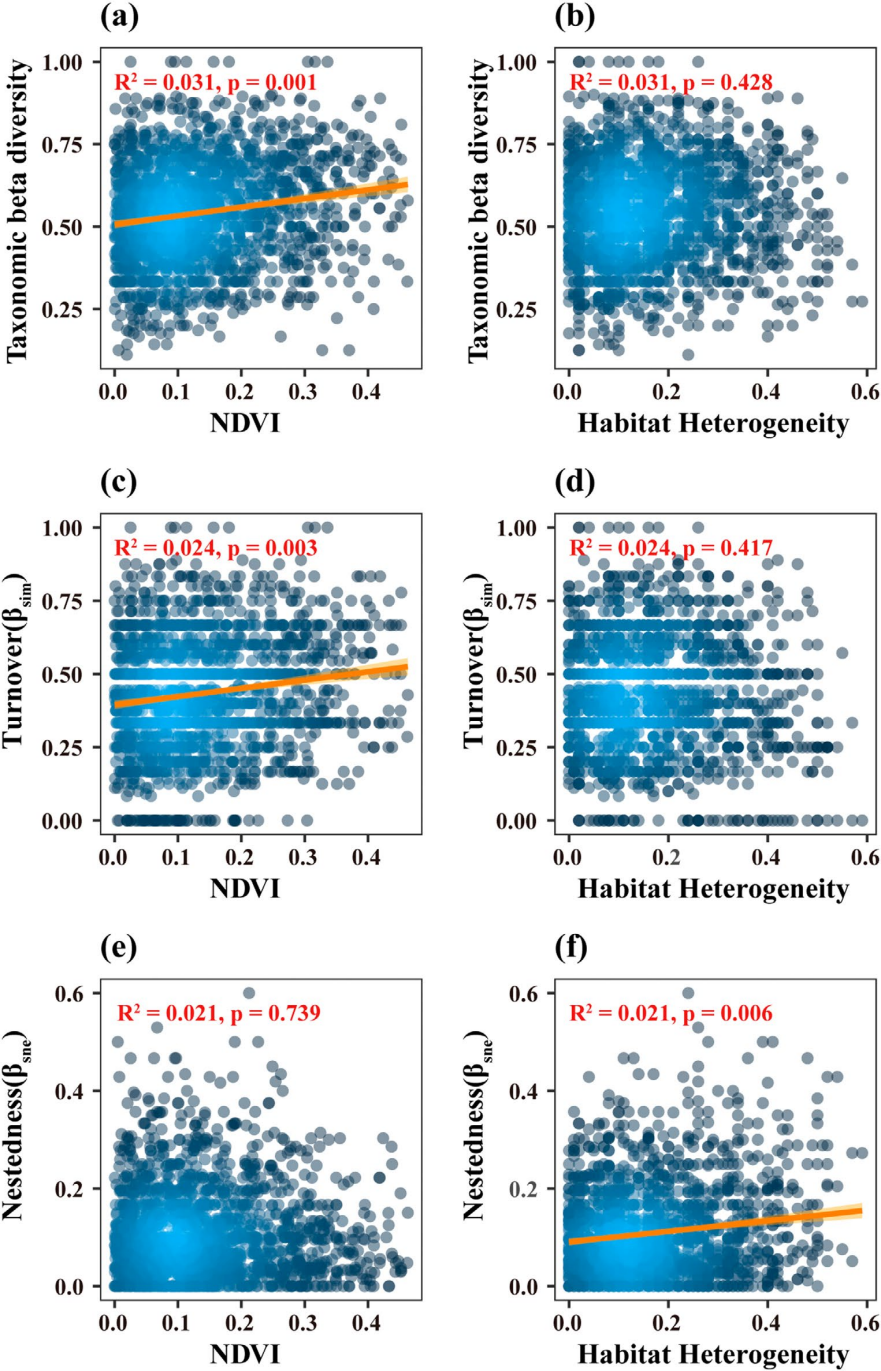
Density scatter plots of summer taxonomic beta diversity (β_sor_) and its two components, species turnover (β_sim_) and nestedness (β_sne_), against pairwise differences in NDVI and habitat heterogeneity, based on Mantel test‐based regression modeling (MRM). The red fitted lines represent the linear regressions derived from the MRM analysis. (a) Total taxonomic beta diversity vs. NDVI. (b) Total taxonomic beta diversity vs. habitat heterogeneity. (c) Turnover component vs. NDVI. (d) Turnover component vs. habitat heterogeneity. (e) Nestedness component vs. NDVI. (f) Nestedness component vs. habitat heterogeneity.

**FIGURE 4:**
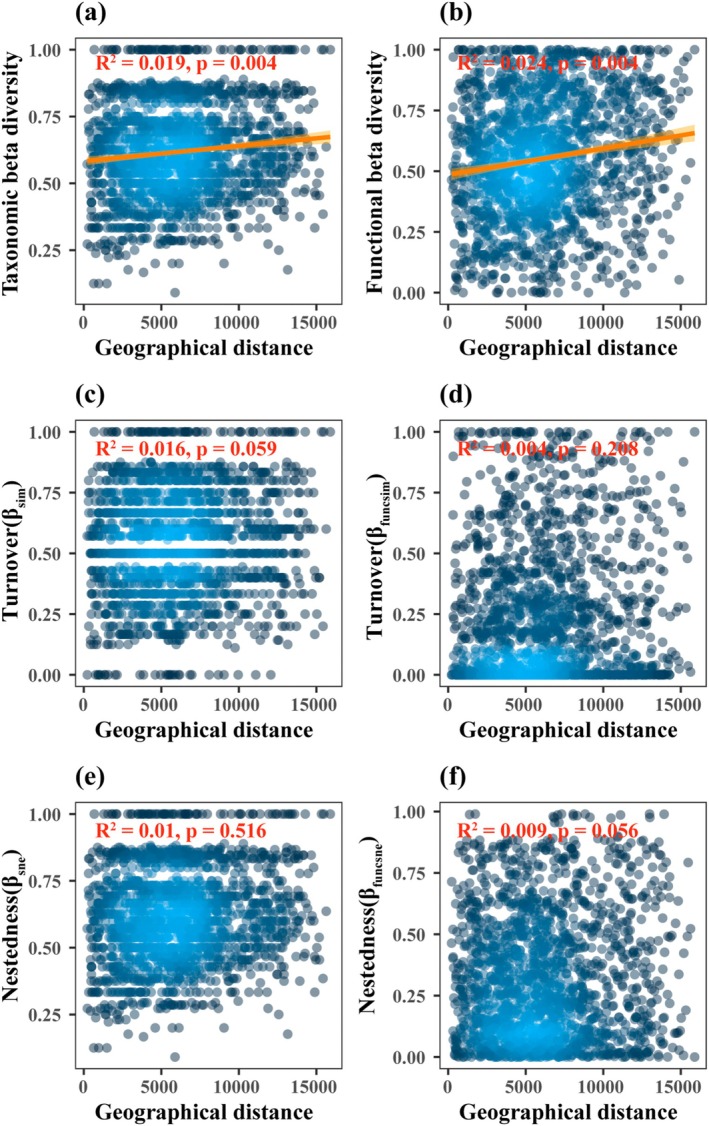
Density scatter plots of winter taxonomic beta diversity (β_sor_) and functional beta diversity (β_funcsor_) against geographic distance, based on Mantel test‐based regression modeling (MRM). The red fitted lines represent the linear regressions derived from the MRM analysis. (a) Total taxonomic beta diversity. (b) Total functional beta diversity. (c) Taxonomic turnover. (d) Functional turnover. (e) Taxonomic nestedness. (f) Functional nestedness.

In addition, the taxonomic beta diversity (β_sor_) and species turnover components (β_sim_) of summer bird communities were significantly positively related with the pairwise difference in NDVI between sites (Figure 3a,c and Table 1), while nestedness components (β_sne_) were significantly positively related with the pairwise difference in habitat heterogeneity between sites (Figure 3f and Table 1). Neither taxonomic beta diversity nor functional beta diversity in summer was related with geographic distance. In winter, no significant relationships were detected between beta diversity and pairwise NDVI differences or pairwise habitat heterogeneity differences. For geographic distance, no significant relationships were found for most beta diversity components, except that geographic distance showed a positive relation with overall winter taxonomic beta diversity and overall functional beta diversity (Figure 4 and Table 1).

**TABLE 1:** The relationship between beta diversity and its components with NDVI, geographical distance, and habitat heterogeneity. In bold, data for significant factors.

		*F*	*R* ^2^	*p*	Coef.	*p*
**Summer**						
β_sor_	NDVI	19.092	0.031	**0.002**	0.263	**0.001**
	Habitat heterogeneity				0.047	0.428
	Geographical distance				−1.792	0.920
β_sim_	NDVI	14.750	0.024	**0.011**	0.279	**0.003**
	Habitat heterogeneity				−0.061	0.417
	Geographical distance				0.369	0.874
β_sne_	NDVI	12.662	0.021	**0.023**	−0.016	0.739
	Habitat heterogeneity				0.108	**0.006**
	Geographical distance				−5.489	0.648
β_funcsor_	NDVI	3.151	0.005	0.761	0.004	0.981
	Habitat heterogeneity				0.141	0.345
	Geographical distance				−0.246	0.602
β_funcsim_	NDVI	0.629	0.001	0.945	0.038	0.775
	Habitat heterogeneity				−0.051	0.624
	Geographical distance				0.052	0.880
β_funcsne_	NDVI	6.180	0.010	0.292	−0.033	0.818
	Habitat heterogeneity				0.192	0.089
	Geographical distance				−0.297	0.407
**Winter**						
β_sor_	NDVI	11.476	0.019	0.298	−0.0004	0.995
	Habitat heterogeneity				0.104	0.101
	Geographical distance				0.594	**0.004**
β_sim_	NDVI	9.551	0.016	0.094	−0.075	0.547
	Habitat heterogeneity				0.155	0.073
	Geographical distance				0.512	0.059
β_sne_	NDVI	9.758	0.016	0.213	0.074	0.210
	Habitat heterogeneity				−0.052	0.186
	Geographical distance				0.824	0.516
β_funcsor_	NDVI	14.742	0.024	0.086	−0.111	0.547
	Habitat heterogeneity				−0.008	0.951
	Geographical distance				0.108	**0.004**
β_funcsim_	NDVI	2.200	0.004	0.584	−0.032	0.853
	Habitat heterogeneity				0.044	0.720
	Geographical distance				0.357	0.208
β_funcsne_	NDVI	5.047	0.009	0.404	−0.079	0.544
	Habitat heterogeneity				−0.051	0.567
	Geographical distance				0.726	0.056

**FIGURE 5:**
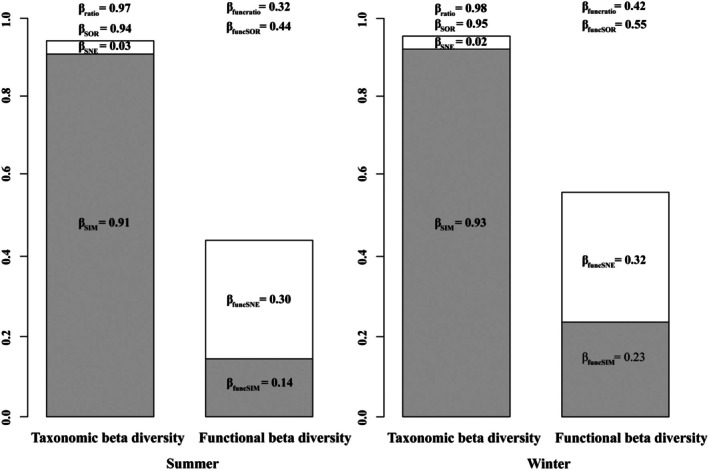
The multi‐site Sørensen dissimilarities (β_SOR_, β_funcSOR_) and their turnover (β_SIM_, β_funcSIM_) and nestedness (β_SNE_, β_funcSNE_) components for bird communities across 60 sites in summer and winter. The beta ratio (β_ratio_, β_funcratio_) denotes the ratio of turnover to overall dissimilarity (β_SIM_/β_SOR_ and β_funcSIM_/β_funcSOR_).

For total taxonomic beta‐diversity in both summer and winter, turnover contributed bigger than nestedness did. Contrastly, for total functional beta diversity, nestedness contributed more (Figure 5). The NODF analysis revealed the presence of nested distribution patterns at the taxonomic level in summer and winter, but no nested patterns at the functional level (Data S6).

## 4 Discussion

Our results show that winter taxonomic alpha diversity of birds is significantly negatively related with habitat heterogeneity, contradicting the classic habitat heterogeneity hypothesis (MacArthur and MacArthur 1961). Although higher habitat heterogeneity is often associated with greater overall resource diversity, in winter the availability of highenergy food (e.g., crop residues, anthropogenic subsidies) is typically higher in more homogeneous, open landscapes such as agri‐cultural fields or village outskirts, rather than in complex heterogeneous patches (Revilla‐Martin et al. 2023). The winter bird community in this region is dominated by a few cold‐tolerant, generalist species (e.g., Eurasian Tree Sparrow *Passer montanus* , Oriental Magpie *Pica serica*), which are not restricted by habitat type when selecting habitats and instead choose sites based solely on resource availability. For these generalists, “resource availability” in winter means easy access to concentrated, high‐energy food sources, which are often found in homogeneous habitats, not in heterogeneous ones (Hancock and Wilson 2003). Winter in this area is cold, dry, and relatively resource‐poor; environmental complexity may not represent greater resource abundance. Habitats mixed with agricultural land, impervious surfaces, and other patch types may provide fewer available resources than pure woodland patches, thereby reducing bird abundance and species richness (Dong et al. 2025). Moreover, highly heterogeneous landscapes often contain more edge habitat, more fragmented patches, and more complex vertical structures, which increase birds' energy expenditure during foraging and movement and expose them to higher predation risk (Tryjanowski et al. 2015), leading to declines in bird abundance and species richness within such areas. Notably, winter functional alpha diversity does not simply decrease linearly with increasing habitat heterogeneity; instead, it exhibits a non‐linear pattern of first increasing and then decreasing, which also deviates from our hypothesis of a linear positive relation. This indicates that under low to moderate heterogeneity, birds with different functional traits can still exploit complementary microhabitat resources, thereby enhancing functional diversity. However, when heterogeneity becomes too high and patches are overly fragmented, functional traits that require large, continuous habitats (e.g., species active in tall tree canopies, waders requiring open water) are lost locally, causing functional diversity to decline (Yang et al. 2023). This non‐linear relationship echoes the intermediate disturbance hypothesis (McKinney 2008). It should be noted that this study focused on horizontal habitat heterogeneity, using the proportion of different patch types as a proxy, and did not directly quantify vertical vegetation structural heterogeneity. Vertical heterogeneity may also influence birds' energy expenditure and concealment conditions in winter; future research should further explore its role in avian diversity within small protected areas during the winter season.

In summer, overall beta diversity and its turnover component are positively related with pairwise differences in NDVI, whereas the nestedness resultant component of beta diversity is positively related with pairwise differences in habitat heterogeneity. In contrast, patches with higher habitat heterogeneity usually contain a greater diversity of habitat types and thus support larger local species pools; patches with lower heterogeneity represent subsets of the larger patches, forming a nested pattern. In conservation practice, maintaining highly heterogeneous patches in summer helps preserve more complete species assemblages, whereas preserving the NDVI gradient benefits the spatial differentiation of species.

In winter, both overall taxonomic beta diversity and functional beta diversity show a significant positive relation with spatial distance whereas differences in NDVI and habitat heterogeneity are not significantly related to any component of winter beta diversity. This contradicts our hypothesis that the turnover component increases with increasing differences in productivity or habitat heterogeneity. This indicates that differences among bird communities in winter are primarily driven by dispersal limitation rather than by environmental gradients. In summer, when resources are abundant and birds have large activity ranges, they can selectively inhabit areas across broad environmental gradients; thus, environmental filtering (productivity, habitat heterogeneity) becomes the main force shaping community assembly. However, winter is characterized by low temperatures, food scarcity, and short day length; birds have limited means of acquiring energy, resulting in reduced individual activity ranges and lower dispersal capacity (Chen et al. 2025; Xu et al. 2025). Under such conditions, the greater the distance between patches, the more difficult it is for individual birds to move among patches, leading to stochastic differences in community composition that increase with distance. Therefore, in the case of a protected area with limited extent, in addition to paying attention to internal habitat quality, it is essential to strengthen spatial connectivity with surrounding key habitats to ensure the effective maintenance of ecological functions of animal communities.

## 5 Conclusion

Our findings reveal productivity, as an important environmental variable, influences bird species richness and compositional differences from two dimensions. A homogeneous forest is more conducive to maintaining the diversity of bird communities than disturbed complex habitats. Even if there is a high level of species turnover in the region, the functions of these species may be homogeneous and redundant, which is not beneficial to improve ecosystem resilience. We need to protect existing forest resources and restore the integrity of the local ecosystem. Several limitations should be noted. First, our beta diversity analyses were based on species presence‐absence rather than abundance data, which may underestimate turnover and nestedness components. Second, we lacked spring and autumn bird data, so our results do not capture migratory dynamics.

## Author Contributions

**Xiaocui Ma:** data curation (equal), formal analysis (equal), methodology (equal), visualization (equal), writing – original draft (equal). **Meiting Wang:** visualization (equal), writing – review and editing (equal). **Shiguang Zhang:** writing – review and editing (equal). **Yiting Jiang:** conceptualization (lead), funding acquisition (equal), methodology (equal), supervision (lead), writing – review and editing (equal). **Dongmei Wan:** conceptualization (equal), funding acquisition (lead), project administration (lead), resources (lead).

## Funding

This work was supported by the National Natural Science Foundation of China (No. 31872231), the Shenyang Science and Technology Bureau Special Project for Scientific and Technological Innovation Talent Cultivation (No. RC230232), and the Xingliao Talents Program (No. XLYC2503182).

## Conflicts of Interest

The authors declare no conflicts of interest.

## Supporting information


**Figure S1:** Species accumulation curves in summer and winter (*n* = 60).
**Table S1:** Bird survey directory, ‘+’represents birds surveyed in spring or winter, ‘−’ represents birds not surveyed in spring or winter.
**Table S2:** Sample coverage of 60 survey sites in summer and winter.

## Data Availability

The data supporting the findings of this study are available within the article and its Supporting Materials. These specifically include: (1) the processed winter and summer bird community composition data (Data S1 and S2) used in this study, (2) the corresponding bird fu trait data for both seasons (Data S3 and S4), (3) the integrated site‐by‐environmental variable matrix derived for this analysis (Data S5), and (4) the NODF results for taxonomic and functional nestedness (Data S6).
