# Supplementary material for: Determinants of Bird Richness in the Louzishan National Nature Reserve: Effects of Productivity and Habitat Heterogeneity Across Taxonomic and Functional Dimensions
Source: Ecol Evol. 2026 Jun 17;16(6):e73860. doi: 10.1002/ece3.73860 (PMC13274549; doi:10.1002/ece3.73860)
Supplement: Supplementary file 1 — Figure S1: Species accumulation curves in summer and winter (n = 60). Table S1: Bird survey directory, ‘+’represents birds surveyed in spring or winter, ‘−’ represents birds not surveyed in spring or winter. Table S2: Sample coverage of 60 survey sites in summer and winter. [file ECE3-16-e73860-s001.pdf]

Figure S1 Species accumulation curves in summer and winter (n = 60).

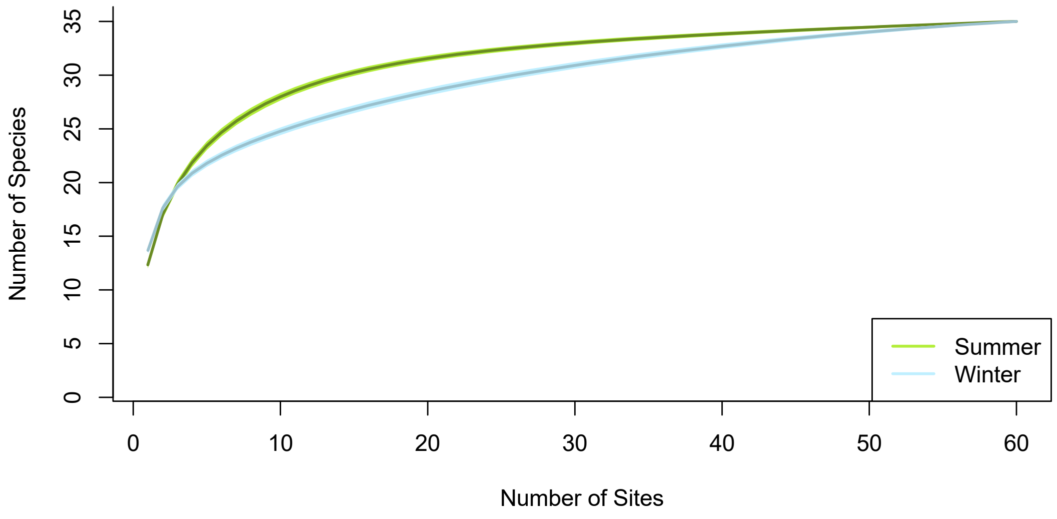

Table S1 Bird survey directory, '+' represents birds surveyed in spring or winter, '-' represents birds not surveyed in spring or winter.

Order	Family	Genus	Species name	Latin name	Spring	Winter
Galliformes	Phasianidae	Phasianus	Ring-necked Pheasant	*Phasianus colchicus*	+	+
Accipitriformes	Accipitridae	Accipiter	Eurasian Sparrowhawk	*Accipiter nisus*	-	+
Columbiformes	Columbidae	Streptopelia	Oriental Turtle-Dove	*Streptopelia orientalis*	+	-
	Columbidae	Streptopelia	Eurasian Collared-Dove	*Streptopelia decaocto*	+	+
	Columbidae	Spilopelia	Spotted Dove	*Spilopelia chinensis*	+	+
Cuculiformes	Cuculidae	Cuculus	Indian Cuckoo	*Cuculus micropterus*	+	-
Piciformes	Picidae	Dendrocopos	Great Spotted Woodpecker	*Dendrocopos major*	+	+
	Picidae	Picus	Grey-headed Woodpecker	*Picus canus*	+	+
Falconiformes	Falconidae	Falco	Lesser Kestrel	*Falco naumanni*	-	+
	Falconidae	Falco	Eurasian Kestrel	*Falco tinnunculus*	+	+
Passeriformes	Laniidae	Lanius	Chinese Grey Shrike	*Lanius sphenocercus*	-	+
	Oriolidae	Oriolus	Black-naped Oriole	*Oriolus chinensis*	+	-
	Corvidae	Garrulus	Eurasian Jay	*Garrulus glandarius*	-	+
	Corvidae	Cyanopica	Azure-winged Magpie	*Cyanopica cyanus*	-	+
	Corvidae	Urocissa	Red-billed Blue Magpie	*Urocissa erythroryncha*	+	+
	Corvidae	Pica	Oriental Magpie	*Pica serica*	+	+
	Corvidae	Corvus	Carrion Crow	*Corvus corone*	+	+
	Corvidae	Corvus	Large-billed Crow	*Corvus macrorhynchos*	+	+
	Paridae	Periparus	Yellow-bellied Tit	*Periparus venustulus*	+	-
	Paridae	Poecile	Marsh Tit	*Poecile palustris*	+	+
	Paridae	Parus	Great Tit	*Parus major*	+	+
	Alaudidae	Alaudala	Asian Short-toed Lark	*Alaudala cheleensis*	-	+
	Pycnonotidae	Pycnonotus	Light-vented Bulbul	*Pycnonotus sinensis*	+	-
	Hirundinidae	Hirundo	Barn Swallow	*Hirundo rustica*	+	-

	Scotocercidae	Horornis	Manchurian Bush Warbler	*Horornis canturians*	+	-
	Aegithalidae	Aegithalos	Silver-throated Bushtit	*Aegithalos glaucogularis*	+	+
	Phylloscopidae	Phylloscopus	Eastern Crowned Warbler	*Phylloscopus coronatus*	+	-
	Leiothrichidae	Pterorhinus	Pere David's Laughingthrush	*Pterorhinus davidi*	+	+
	Paradoxornithidae	Rhopophilus	Beijing Babbler	*Rhopophilus pekinensis*	+	+
	Paradoxornithidae	Suthora	Vinous-throated Parrotbill	*Suthora webbiana*	+	+
	Zosteropidae	Zosterops	Swinhoe's White-eye	*Zosterops simplex*	+	-
	Regulidae	Regulus	Goldcrest	*Regulus regulus*	-	+
	Sittidae	Sitta	Snowy-browed Nuthatch	*Sitta villosa*	+	+
	Turdidae	Turdus	Grey-backed Thrush	*Turdus hortulorum*	+	-
	Turdidae	Turdus	Chinese Blackbird	*Turdus mandarinus*	-	+
	Turdidae	Turdus	Red-throated Thrush	*Turdus ruficollis*	-	+
	Turdidae	Turdus	Naumann's Thrush	*Turdus naumanni*	-	+
	Muscicapidae	Ficedula	Yellow-rumped Flycatcher	*Ficedula zanthopygia*	+	-
	Muscicapidae	Phoenicurus	Daurian Redstart	*Phoenicurus auroreus*	+	-
	Passeridae	Passer	Eurasian Tree Sparrow	*Passer montanus*	+	+
	Prunellidae	Prunella	Siberian Accentor	*Prunella montanella*	-	+
	Motacillidae	Dendronanthus	Forest Wagtail	*Dendronanthus indicus*	+	-
	Fringillidae	Carpodacus	Pallas's Rosefinch	*Carpodacus roseus*	-	+
	Fringillidae	Chloris	Oriental Greenfinch	*Chloris sinica*	+	+
	Emberizidae	Emberiza	Godlewski's Bunting	*Emberiza godlewskii*	+	+
	Emberizidae	Emberiza	Meadow Bunting	*Emberiza cioides*	+	+
	Emberizidae	Emberiza	Little Bunting	*Emberiza pusilla*	-	+
	Emberizidae	Emberiza	Rustic Bunting	*Emberiza rustica*	-	+
	Emberizidae	Emberiza	Yellow-throated Bunting	*Emberiza elegans*	+	-

Table S2 Sample coverage of 60 survey sites in summer and winter.

Survey point	Summer sample coverage (%)	Winter sample coverage (%)
1	73	93
2	69	85
3	96	100
4	83	97
5	76	96
6	72	82
7	65	66
8	78	100
9	89	100
10	90	100
11	78	100
12	84	97
13	93	79
14	91	97
15	81	88
16	85	88
17	88	96
18	83	98
19	100	88
20	86	74
21	96	88
22	93	95
23	96	94
24	92	89
25	83	93
26	96	94
27	83	91
28	65	92
29	97	86
30	85	100
31	95	100
32	84	90
33	92	97
34	93	96
35	79	99
36	100	96
37	75	98
38	81	81
39	100	83

40	100	91
41	100	98
42	90	78
43	70	97
44	91	70
45	86	91
46	98	91
47	100	98
48	98	97
49	93	67
50	67	85
51	84	100
52	90	88
53	96	88
54	96	94
55	91	99
56	92	98
57	100	72
58	94	98
59	90	98
60	94	97
